# B lymphocytes as direct antigen-presenting cells for anti-tumor DNA vaccines

**DOI:** 10.18632/oncotarget.12178

**Published:** 2016-09-21

**Authors:** Viswa Teja Colluru, Douglas G. McNeel

**Affiliations:** ^1^ Department of Medicine, University of Wisconsin-Madison, Madison, WI, USA; ^2^ Carbone Cancer Center, University of Wisconsin-Madison, Madison, WI, USA

**Keywords:** DNA vaccine, B cells, dendritic cells, direct presentation, Immunology and Microbiology Section, Immune response, Immunity

## Abstract

In spite of remarkable preclinical efficacy, DNA vaccination has demonstrated low immunogenicity in humans. While efforts have focused on increasing cross-presentation of DNA-encoded antigens, efforts to increase DNA vaccine immunogenicity by targeting direct presentation have remained mostly unexplored. In these studies, we compared the ability of different APCs to present antigen to T cells after simple co-culture with plasmid DNA. We found that human primary peripheral B lymphocytes, and not monocytes or *in vitro* derived dendritic cells (DCs), were able to efficiently encode antigen mRNA and expand cognate tumor antigen-specific CD8 T cells *ex vivo*. Similarly, murine B lymphocytes co-cultured with plasmid DNA, and not DCs, were able to prime antigen-specific T cells *in vivo.* Moreover, B lymphocyte-mediated presentation of plasmid antigen led to greater Th1-biased immunity and was sufficient to elicit an anti-tumor effect *in vivo*. Surprisingly, increasing plasmid presentation by B cells, and not cross presentation of peptides by DCs, further augmented traditional plasmid vaccination. Together, these data suggest that targeting plasmid DNA to B lymphocytes, for example through transfer of *ex vivo* plasmidloaded B cells, may be novel means to achieve greater T cell immunity from DNA vaccines.

## INTRODUCTION

Recent scientific advancements point to an indispensable role of the adaptive immune system in regulating cancer progression and therapy, in spite of being a malady of the “self” [1, 2]. The significant clinical successes of checkpoint blockade to simply remove the “brakes” from T cells further highlight the potential role of therapeutic adaptive immune recognition in cancer [3]. While a subset of patients responds readily to checkpoint blockade, there exists a large non-responder patient population [4]. The decisive factor that determines whether a patient will respond to these highly effective immunotherapies seems to be the presence of an adequate T cell response to tumor antigens [5]. The primary method to elicit and/or augment T cell immunity to date remains vaccination - either targeting specific antigens or whole tumor cells. In spite of encouraging preclinical data for multiple types of anti-tumor vaccines, clinical successes remain few [6]. To date, there has been only one U.S. FDA-approved vaccine as a treatment for cancer, Sipuleucel-T (Provenge^®^), for patients with castration resistant metastatic prostate cancer [7]. One of the primary limitation for anti-tumor vaccines in human trials remains the relatively low immunogenicity when compared to preclinical models. Of the antigen-specific vaccine modalities explored (including RNA, DNA, *Listeria,* and synthetic long peptides (SLP)) with the potential to induce robust CD4 and CD8 T cell responses *in vivo*, DNA vaccines are notorious for their low immunogenicity in human trials [8, 9].

DNA vaccines are simple bacterial plasmids encoding the antigen of interest, most commonly under the control of a strong viral promoter [8, 10]. Based on observations that simple inoculation of naked plasmid DNA could lead to transgene expression in muscle tissue *in vivo* [11]*,* and that such transgene expression was immunogenic [12, 13], DNA vaccines have been extensively evaluated in preclinical models of infectious and malignant disease [8]. In spite of remarkable preclinical success, immune response upon DNA vaccination remains modest in human trials [9]. Investigations into the mechanisms of DNA vaccine immunogenicity led to the surprising finding that even though transfection of a small number of dendritic cells (DC) occurs after *in vivo* DNA administration [14-17], they have little relevance to the generation of *de novo* immune responses upon vaccination [18-22]. Notably, most of the immunogenicity relied on production of the antigen in bystander skin or muscle cells, and subsequent “cross presentation” of this antigen by antigen presenting cells (APCs). As such, there is little “direct presentation” involved in which there is cell intrinsic activation and antigen presentation by a professional APC.

While incrementally successful efforts to improve DNA vaccine immunogenicity have largely focused on increasing the amount of antigen delivered *in vivo* through increasing (1) transfection efficiency [23-25]** and (2) optimization of the plasmid vector [10, 26, 27], these techniques act primarily by enhancing cross presentation of antigen [22]. A relatively unexplored avenue of investigation is to determine whether the immunogenicity of DNA vaccines might be increased by augmenting direct presentation. Most recent efforts have focused on augmenting DC recruitment and presentation, through targeting of the antigen to DCs or recruitment of myeloid APC subsets [28-30]. However, efforts to employ DC or monocyte promoters in DNA vaccines have yielded mixed results, [18, 20-22, 31]. Other investigators have conversely reported that B lymphocytes are able to spontaneously encode and present antigen upon co-incubation with plasmid DNA harboring an IgG promoter [32-34]. In the studies described herein, we sought to identify the APC types best able to directly present antigens encoded by plasmid DNA vaccines, and examine their effect on DNA vaccine immunogenicity *in vivo*.

Using primary B cells, monocytes/macrophages, and DCs, our studies demonstrate that human B cells are able to encode antigen mRNA and expand cognate antigen specific CD8 T cells upon simple co-culture with plasmid DNA. Moreover, only murine B cells, and not DCs, were able to prime an immune response *in vivo* that resulted in an anti-tumor effect. In addition, supplementing traditional DNA vaccination with B cells loaded *ex vivo* with plasmid DNA led to greater antigen specific CD8 T cell proliferation *in vivo.* Together these results suggest that targeted delivery of DNA to B cells as cells capable of direct presentation may be a preferred means to augment the anti-tumor efficacy of DNA vaccines.

## RESULTS

### Primary human peripheral blood APCs exhibit spontaneous uptake of plasmid DNA

In order to characterize spontaneous uptake of plasmid DNA by different primary APCs, we utilized mixed populations of autologous cells and fluorescently labeled plasmid DNA. To ensure a full complement and sufficient cell numbers of each of the different professional APC types of interest, namely, monocytes/macrophages, dendritic cells (DC), and B lymphocytes, we added autologous monocyte-derived dendritic cells (CD14^−^ CD11c^+^ MHC-II^hi^) to peripheral blood mononuclear cells (PBMCs). To control for possible changes to the DNA structure by labeling, plasmid DNA was covalently labeled with either a Cy5 fluorophore dye or using a fluorescently-labeled peptide nucleic acid (PNA) sequence-specific probe (data not shown).

**Figure 1:**
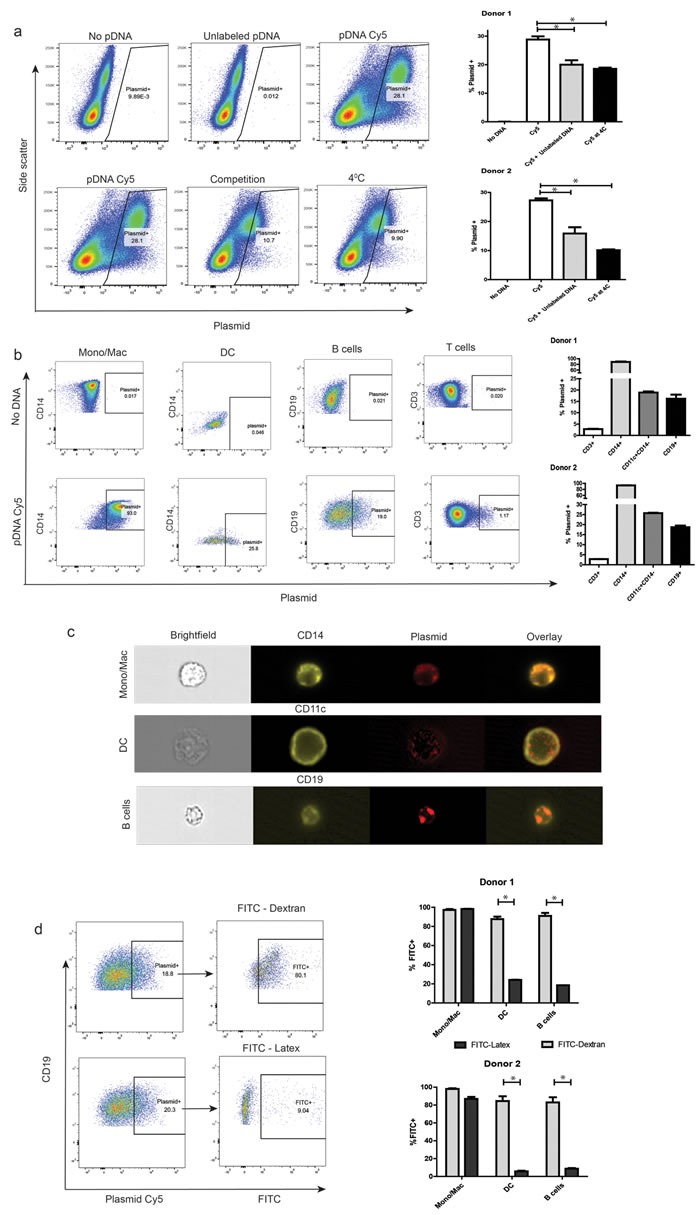
Primary human peripheral blood APCs exhibit spontaneous uptake of plasmid DNA **a.** Autologous monocyte-derived DCs were added to cryopreserved human PBMCs. Cells were washed and re-suspended in PBS. Covalently labeled plasmid DNA was then added to samples that were either pre-incubated at 37°C or 4°C, both alone or along with 5μg/mL unlabeled DNA for 1hr, washed, and analyzed by flow cytometry. Dot plots of live single cells in each of the conditions are represented, with side-scatter and plasmid-associated fluorescence on the axes (left). Plasmid-associated fluorescence fractions in the different conditions for two representative donors (right). **b.** Cells were treated as above, and then stained for relevant surface markers before flow cytometric analysis. Subset-wise dot-plot representation of plasmid-associated fluorescence from a representative donor (left). Data on subset-wise association from two representative donors (right). **c.** Samples were treated as above, and analyzed for internalization of plasmid by imaging cytometry at a 60X magnification. Shown is a representative cell image of each APC cell type with internalized plasmid. **d.** Cells were treated as above, in addition to co-incubation with either FITC-Dextran or FITC-Latex beads. Plasmid^+^ APC sub-populations were then assayed for their association with either reagent. Representative dot plots showing extent of pinocytosis (FITC-Dextran^+^, left-top) and phagocytosis (FITC-Latex^+^, let-bottom) in plasmid positive B cells. The right panel is a graphical representation of data from similar studies utilizing cells from 2 separate donors. For all panels, * denotes a *p*-value <0.05, two-sided t-test. Data is representative of at least 5 independent experiments, involving at least 5 separate donors. Error bars represent mean + SEM.

DC-enriched PBMCs were incubated in the presence of 2μg/mL fluorescently-labeled plasmid DNA. As shown in Figure 1a (left) there was robust association of fluorescent plasmid with primary human PBMC after just 1h, with greater than 25% of cells positive for association/uptake of DNA. This was significantly reduced upon competition with 5μg/mL unlabeled plasmid DNA or incubation of cells at 4°C, suggesting that cells were exhibiting plasmid DNA uptake through an active mechanism. A graphical representation of these data is as shown in Figure 1a (right). As expected, plasmid uptake was robust in the different professional APC types, and less in the T lymphocyte fraction (Figure 1b). Strikingly, nearly all of the lineage^+^ myeloid mononuclear cells exhibited plasmid association, consistent with their highly phagocytic nature. DCs and B lymphocytes exhibited moderate association, with ∼25% of the cells gating positive for Cy5. A graphical representation of these data from two of five donors is as shown in Figure 1b (right). To confirm that plasmid-associated fluorescence was indicative of uptake and internalization, cells were treated as above and Cy5+ events were further analyzed using multispectral imaging cytometry. As seen in representative images in Figure 1c, all Cy5+ APC types exhibited internalization of plasmid. Quantification revealed internalization of fluorescence on greater than 90% of Cy5+ events in each cell type (Figure S1). We further identified the B cell sub-population exhibiting uptake as mature naïve, IgD^+^CD27^−^CD19^+^ cells (Figure S2).

To investigate the mechanism of plasmid uptake in the different APC types, we marked cells exhibiting pinocytosis or phagocytosis using FITC-dextran and FITC-labeled latex beads (FITC-latex), respectively. Samples were treated with Cy5 labeled plasmid DNA as above, and additionally incubated with either FITC-Dextran or FITC-latex. As expected, all monocytes/macrophages were positive for both FITC-Dextran and FITC-latex, given their high phagocytic capacity (Figure 1d, right). However, plasmid positive DCs and B cells were each associated with cells exhibiting pinocytosis as marked by FITC dextran, rather than phagocytosis as marked by FITC-latex (Figure 1d, right). This suggests that plasmid DNA is preferentially taken up through fluid phase pinocytosis, at least by DCs and B cells.

### Primary human B cells encode plasmid mRNA and mediate expansion of cognate tumor antigen-specific CD8 T cells upon plasmid DNA treatment

**Figure 2:**
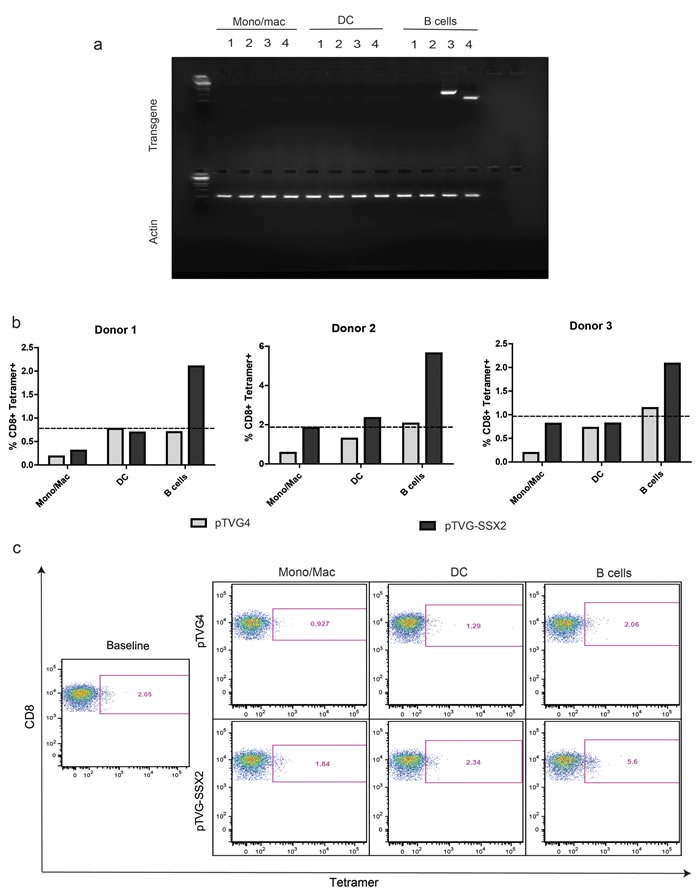
Primary human B cells encode plasmid mRNA and mediate expansion of cognate antigen specific CD8 T cells upon plasmid DNA treatment **a.** Different APC subsets were treated with 25μg/mL of plasmid vector alone, or plasmid DNA encoding either EGFP or ovalbumin (OVA) for 1h as indicated, and then in complete medium for 18h. Total RNA was extracted and assayed for expression of EGFP mRNA by qRT-PCR. Shown is one representative agarose gel of an RT-PCR product. Lanes 1-4 in the top segment represent the following: 1: pTVG4 (empty vector), assay for GFP mRNA; 2: pTVG4, assay for OVA mRNA; 3: pEGFPc1, assay for GFP mRNA; 4: psOVA, assay for OVA mRNA. All lanes in the bottom segment represent assay for the actin housekeeping gene mRNA from the corresponding samples. **b.** Enriched APC subsets from HLA-A2+ donors with known tetramer responses to SSX2 were treated with 25μg/mL empty vector control (pTVG4) or a plasmid encoding SSX2 (pTVG-SSX2) in the presence of autologous T cells. One week later, samples were assayed for p103-specific CD8 T cells by tetramer staining. The dotted line represents baseline *ex vivo* levels of tetramer staining prior to culture. These data are representative of at least 2 independent experiments from each donor. **c.** Representative flow cytometry dot-plots of the results obtained from the different conditions, with tetramer-associated fluorescence on the X axis, and CD8a staining on the Y axis. Data are representative of 4 independent experiments, with at least 3 different donors.

We next examined the ability of different APCs subsets to transcribe plasmid antigen mRNA, and further serve as antigen presenting cells to expand antigen-specific CD8 T cells. DC were prepared as above, and monocyte/macrophage and B cell populations were purified from peripheral blood samples. Enriched cell samples were then cultured with either empty plasmid vector or plasmid DNA encoding enhanced green fluorescent protein (EGFP) or ovalbumin (OVA) prior to extraction of total RNA and analysis for transgene expression by RT-PCR. As seen in Figure 2a, mRNA for EGFP or OVA was detected only in B lymphocytes treated with the respective plasmid construct, and not in dendritic cells or monocytes/macrophages. No detectable levels of transgene protein were observed in any cell type by immunoblot, quantitative ELISA, or flow cytometry (data not shown).

To determine whether specific APC subsets could present DNA-encoded antigens to CD8+ T cells, monocytes/macrophages, DC, and B lymphocytes were enriched from peripheral blood cells of HLA-A2^+^ donors with known pre-existing responses to the dominant epitope (p103) of the tumor-associated antigen, SSX2. Cells were cultured initially with plasmid DNA vector (pTVG4) or plasmid DNA encoding SSX2 (pTVG-SSX2), as above, and then autologous T cells were added. After one week, the frequencies of p103-specific CD8 T cells were assayed by tetramer staining. As shown in Figure 2b, 2c and Table S1, only B lymphocytes treated with pTVG-SSX2, and not the pTVG4 control, were able to expand p103 antigen-specific CD8 T cells *in vitro.* Treatment of B cells with plasmid DNA also resulted in an increase in CD83 and CD86, molecules associated with activation and co-stimulation, on plasmid positive B cells 24h after treatment with plasmid DNA (Figure S3). Taken together, these results demonstrate that following simple co-culture with plasmid DNA, only human B cells act as primary APC by being able to transcribe and present the encoded antigen to expand cognate primary CD8 T cells.

### Plasmid DNA is degraded more rapidly and remains primarily non-nuclear in dendritic cells when compared to B lymphocytes

**Figure 3:**
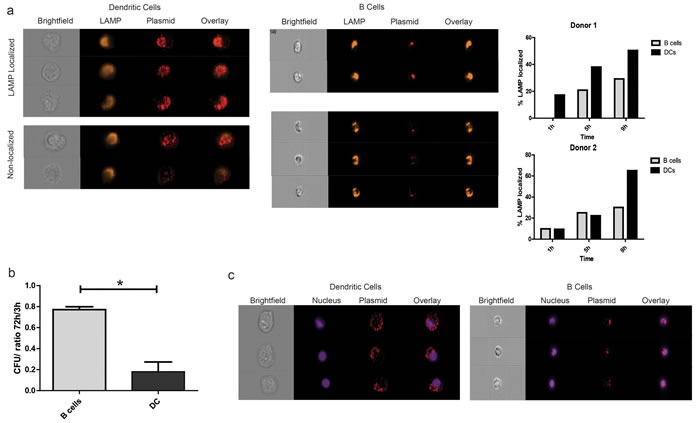
Plasmid DNA is degraded more rapidly and remains primarily cytoplasmic in dendritic cells when compared to B lymphocytes **a.** Enriched APC subsets were treated with fluorescently-labeled plasmid DNA as above and incubated for 1h, 5h, or 9h (1h in PBS, followed by culture in RPMI/10%AB media). Samples were then stained with a live dead marker and an antibody against the lysosome associated membrane protein (LAMP) and analyzed for intracellular localization by imaging cytometry at a 40X objective magnification. Shown are representative images of live CD11c^+^ and CD19^+^ cells that were plasmid positive after 9h that either co-localized with LAMP expression or not (left). Graphical representation of co-localization over time in two different donors as determined by the fraction of cells with a similarity-dilate score > 1 between the relevant fluorescence masks (right). **b.** Enriched APC subsets treated with pTVG4 were cultured for 3h or 72h and subjected to total nucleic acid extraction. Internal plasmid DNA was quantified by transformation of highly competent bacteria. Shown are number of colonies obtained from transformation with 100ng of total internal DNA, normalized to the number of cells of the particular type plated. **c.** Cell subsets were treated as in A, and stained with Hoechst 33342 prior to analysis. Cells with the highest plasmid nuclear localization scores were then retrieved using IDEAS 6.0, and visually verified for translocation into the nucleus. Shown are representative images of dendritic cells and B lymphocytes. Data are representative of 3 separate experiments, with at least 2 different donors.

This finding that monocytes and monocyte-derived DC, despite their ability to take up plasmid DNA following simple co-culture, were unable to encode antigen or present antigen to CD8+ T cells was surprising. Consequently, we sought to determine whether plasmid DNA had different intracellular fates in B lymphocytes and DCs following uptake. First, we examined the association of plasmid DNA with the lysosome associated membrane protein (LAMP) over time in B cells and DCs, as a marker for possible degradation. These cell subsets were isolated as above, and co-incubated with Cy5-labeled plasmid DNA. Samples were analyzed by imaging cytometry. There was clear localization of plasmid DNA to LAMP-stained vesicles in a subset of both cell types, as seen in Figure 3a (left). However, by 9 hours most of the plasmid in DCs had localized to lysosomal vesicles, in contrast to B cells. To determine whether this resulted in plasmid destruction, DC and B cells were treated with plasmid DNA as above by co-culture for either 3h or 72h, and then treated with DNaseI to digest any non-internalized plasmid DNA. Total nucleic acid was subsequently extracted from these samples and used to transform competent bacteria. The number of colonies counted the next day then served as a surrogate for the amount of intact plasmid within the cells. As seen in Figure 3b, the loss of colonies in the DC population over the 3-day period was much greater than the B cells, suggesting progressive intracellular plasmid degradation. Lastly, we studied nuclear trafficking of plasmid DNA within DC and B cell subsets, as a necessary precursor for transcription. Cells were treated with Cy5 labeled plasmid DNA for 9h as above, and stained with Hoechst 33342 to mark the nucleus. Representative images of cells with the highest nuclear localization scores are shown in Figure 3c. As seen in the figure, the majority of the plasmid was restricted to the non-nuclear (perinuclear or cytoplasmic or vacuolar) compartments in DCs as compared to B cells. Together, these results suggest that plasmid DNA, following simple co-culture, is more rapidly degraded in DCs than B lymphocytes through association with the lysosome, with little traffic to the nucleus in DC.

### Mouse B cells, and not dendritic cells, are able to expand antigen specific CD4 and CD8 T cells following DNA stimulation *in vitro*

**Figure 4:**
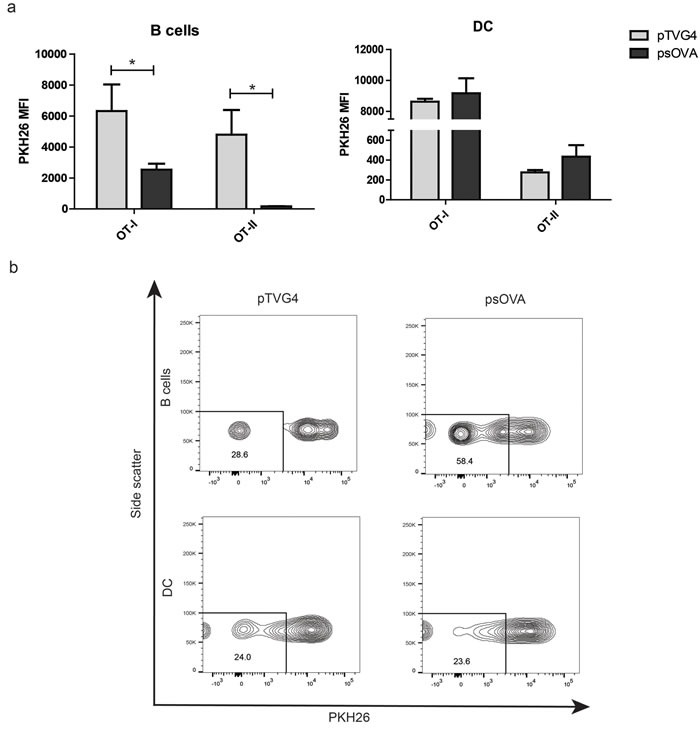
Mouse B cells, and not dendritic cells, are able to expand antigen specific CD4 and CD8 T cells following DNA stimulation **a.** Enriched CD4 and CD8 T cells from OT-II and OT-I transgenic mice were labeled with PKH26 and co-cultured with APC subsets treated with DNA as above (3:1 APC: T cell ratio). One week later, T cells were assayed for loss of PKH26 by flow cytometry. Graphs represent normalized median fluorescence intensity of PKH26 staining on CD8 (left) or CD4 (right) T cells. **b.** Representative histograms from one independent replicate of PKH26 staining under the different conditions for CD8 T cells. For all panels, * denotes a *p*-value < 0.05, two-sided *t*-test. Data are representative of 3 independent experiments. Error bars represent mean + SEM.

We next studied whether murine B cells and DCs similarly differed in their ability to present DNA-encoded antigens upon simple co-incubation. Primary mouse B cells and DCs were obtained and enriched from spleens of wild-type or FLT3-L-treated mice, respectively. OT-I and OT-II TCR transgenic cells were labeled with PKH26, and added to B cells or DC that had been pre-incubated with plasmid DNA encoding ovalbumin. One week later, PKH26 dilution in CD4 and CD8 T cell populations was measured by flow cytometry. As demonstrated in Figure 4, only B lymphocytes treated with a plasmid encoding ovalbumin were able to mediate proliferation of ovalbumin-specific OT-I and OT-II cells, consistent with our findings using human cells.

### Mouse B cells, and not dendritic cells, are able to prime an antigen specific CD8 T-cell response following DNA stimulation *in vivo*

**Figure 5:**
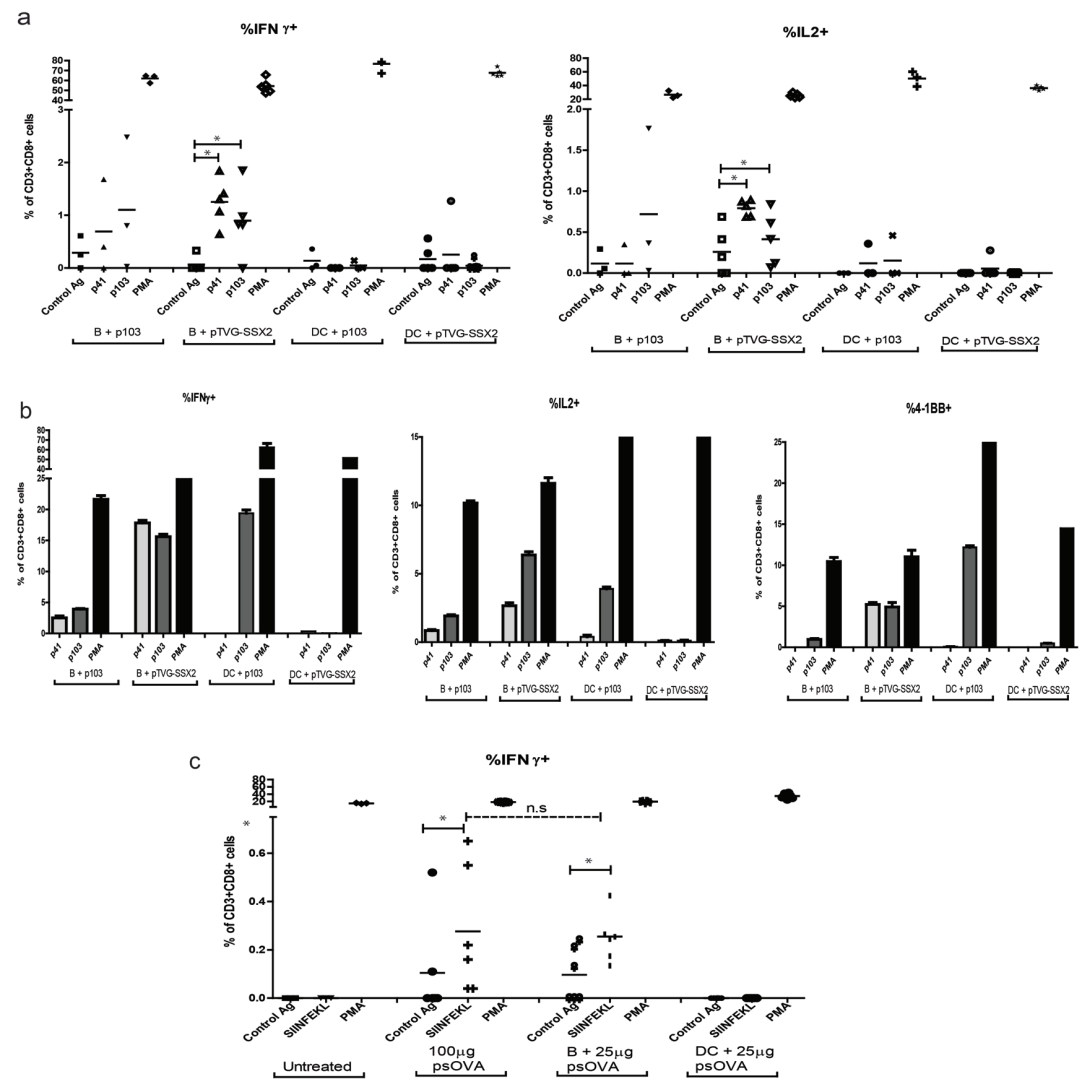
Plasmid treated mouse B cells, and not dendritic cells, are able to prime an antigen-specific CD8 T cell response *in vivo* **a.** Enriched B cells or DCs were obtained from HHDII-DR1 mice and treated with either 2μg/mL of the p103 peptide or 25μg/mL of pTVG-SSX2 DNA and intradermally transferred into naïve HHD-II/DR-I mice (*n* = 6/treatment group). Two weeks later, splenocytes from the different treatment groups were assessed for antigen-specific CD8 T cells by intracellular cytokine staining for IFNγ (left) or IL2 (right) expression following stimulation with control peptide, SSX2-derived p41 or p103 peptides, or PMA/Ionomycin (PMA, positive control). **b.** Pooled splenocytes were stimulated *in vitro* with peptide for one week prior to flow cytometric analysis. Shown are antigen-specific CD8 T cells expressing IFNγ (left), IL2 (center), or CD137/4-1BB (right) following re-stimulation. **c.**. Enriched B cells and DCs were treated with 25μg of psOVA and subcutaneously transferred into naïve C57BL6 mice (*n* = 4-6/group). Another group received 100 μg psOVA alone intradermally. Two weeks later, splenocytes were analyzed for antigen-specific CD8 T cell responses to the SIINFEKL epitope by intracellular cytokine staining following *in vitro* stimulation as above. For all panels, data are corrected for background and * denotes a *p*-value < 0.05, two-sided *t*-test. Data are representative of at least 3 independent experiments. Error bars represent mean + SEM.

We next evaluated the ability of B cells or dendritic cells to serve as direct presenting cells for plasmid DNA *in vivo* to prime an adaptive T-cell response. For these studies we used two model antigens - SSX2 and ovalbumin, on the HLA-A2 transgenic (HHDII-DR1) and the H-2K^b^ backgrounds, respectively. In the first model, B cells and DCs were isolated from the spleens of HHDII-DR1 mice as above and co-incubated with either plasmid DNA encoding SSX2 (pTVG-SSX2) or the p103 HLA-A2-restricted nonamer peptide. After 18h, cells from each treatment type were washed and transferred into groups of naïve syngeneic mice. Importantly, DCs were not matured with GM-CSF after treatment with plasmid DNA or peptide in these studies to facilitate antigen uptake and subsequent maturation [35-37]. Immune responses were assessed two weeks after a single cell transfer. As can be seen in Figure 5a, only transfer of B cells co-cultured with plasmid DNA led to antigen-specific IFNγ and IL2 release by CD8 T cells. Immune responses were further evaluated after a 1-week *in vitro* stimulation, to detect lower frequency responses (Figure 5b). As shown, responses were detectable to both dominant (p103) and subdominant (p41) epitopes following delivery of DNA by B cells, but DC were only able to present the peptide antigen to expand CD8 T cells. CD8 T cells also demonstrated increased CD137 expression, as a marker of antigen-specific activation [38]. In the second model, B cells and DCs were obtained from the spleens of wild type C57/BL6 mice, treated as above by simple co-culture with plasmid DNA encoding ovalbumin or SIINFEKL peptide, and adoptively transferred into naïve age-matched C57BL6 mice. Two weeks later, splenic CD8 T cells were assayed for their ability to secrete IFNγ upon stimulation with the SIINFEKL epitope. Once again, we were able to detect antigen-specific cytokine release only upon B cell presentation of plasmid DNA, and not following DC presentation (Figure 5c). Of note, transfer of B cells co-cultured with even less plasmid DNA (25 μg) was sufficient to elicit the same magnitude of response as observed with traditional intradermal plasmid vaccination using 100 μg DNA (Figure 5c). These data confirm that B lymphocytes, and not dendritic cells, are able to efficiently prime a murine immune response after simple *in vitro* co-culture with plasmid DNA.

### B cells, and not dendritic cells, are able to prime an anti-tumor response that can infiltrate tumors *in vivo* following DNA co-culture

**Figure 6:**
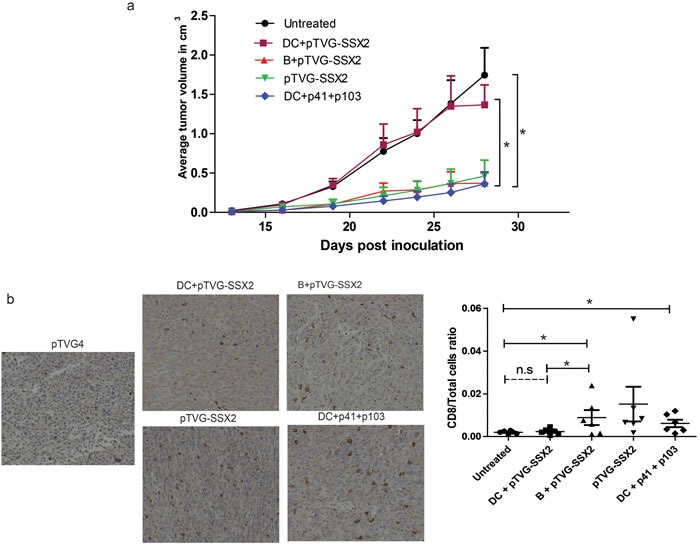
Plasmid treated mouse B cells, and not dendritic cells, are able to prime an anti-tumor response and elicit tumor infiltration of CD8 T cells *in vivo* **a.** B cells and DCs from HLA-A2 expressing mice were obtained as above. B cells were treated with pTVG-SSX2, and DCs were treated with either the pTVG-SSX2 plasmid or the p41/p103 peptides along with 1000U/mL GM-CSF. Cells were cultured for 18h and adoptively transferred into the ear pinna of HHDII-DR1 mice previously inoculated with a syngeneic sarcoma cell line overexpressing SSX2 (*n* = 5-6/group). Tumor size was monitored over time per group. **b.** Representative immunohistochemistry staining for CD8a in the tumor sections from the different groups (left). Tumors were enzymatically digested to obtain single cell suspensions and stained for infiltration of immune cells. The graph on the right represents the CD8 T cell /total live cell ratio of each animal in each treatment group. For all panels, * denotes a *p*-value < 0.05, two-sided *t*-test. Data are representative of at least 2 independent experiments. Error bars represent mean + SEM.

We have previously demonstrated that a DNA vaccine encoding SSX2 can mediate anti-tumor responses in tumor-bearing mice [39]. To evaluate the ability of APC populations primed *ex vivo* with plasmid DNA for their ability to elicit anti-tumor responses, naïve HHDII-DRI mice were inoculated with a syngeneic sarcoma cell line engineered to overexpress SSX2 [39]. Two days and 16 days later, B cells and DCs that had been previously co-cultured with either pTVG-SSX2 plasmid or SSX2-derived p41 and p103 HLA-A2 peptides were administered intradermally. An additional group received plasmid DNA alone, also administered intradermally. As demonstrated in Figure 6, B cells loaded with pTVG-SSX2 DNA, but not DC loaded with DNA, were able to elicit an anti-tumor response. DC were, however, able to elicit an anti-tumor response when loaded with peptide. Groups that had delays in tumor growth had an increase in tumor-infiltrating CD8 T cells as assessed by IHC and flow cytometric analyses (Figure 6b).

### B-cell presentation of DNA-encoded antigens affects Th1 cytokine responses and increases antigen-specific CD8 T cell proliferation *in vivo*

**Figure 7:**
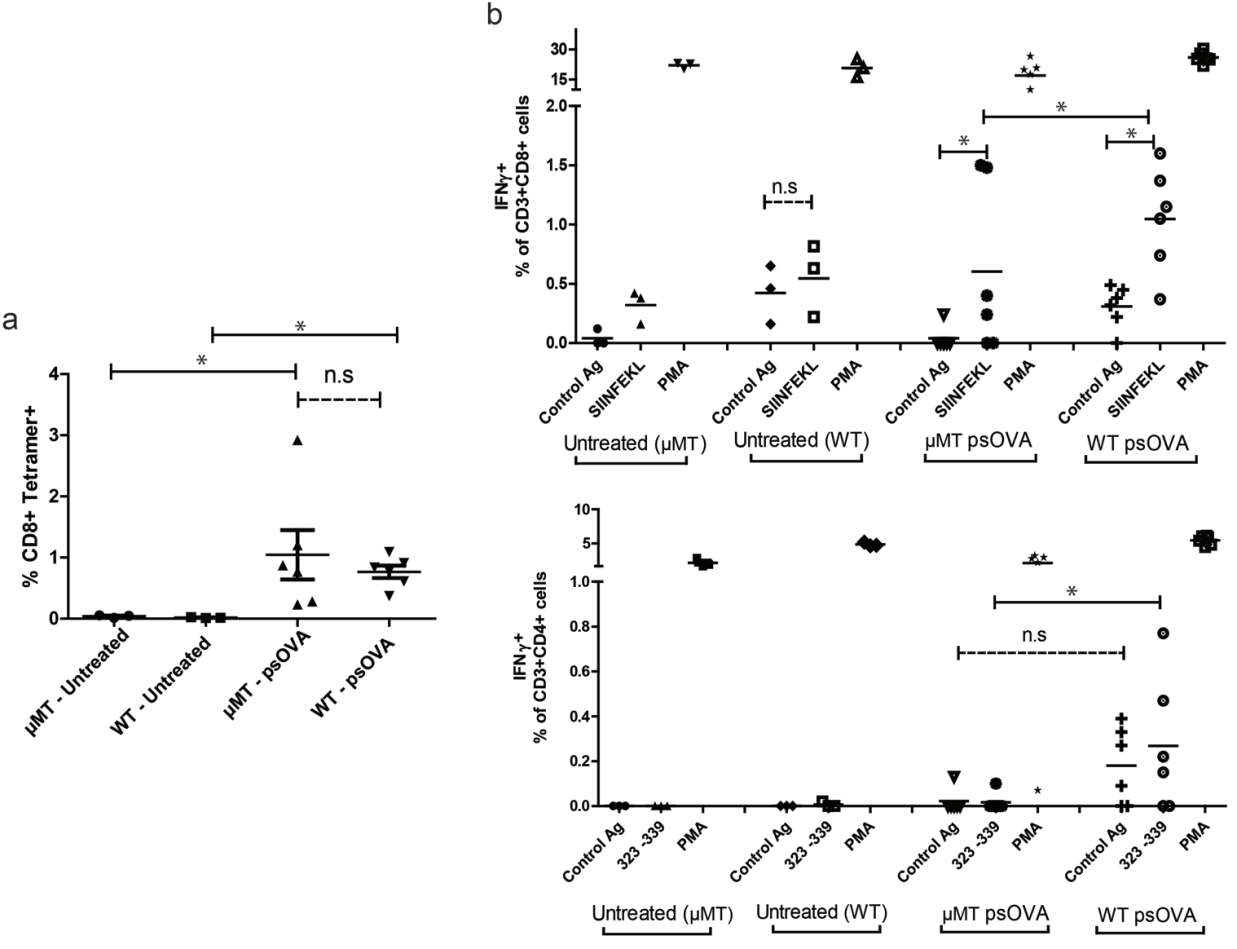
Immunization of B cell-deficient mice results in equivalent frequencies of antigen-specific CD8 T cells as measured by tetramer staining, but lower antigen-specific IFNγ secretion by CD4 and CD8 T cells Age-matched B cell-deficient BL6 μMT mice and wild-type C57BL6 mice (*n* = 6/treatment group) were immunized four times at weekly intervals with 100μg of psOVA by intradermal injection. **a.** One week after the final injection, frequencies of antigen-specific CD8 T cells in the spleen were assayed by SIINFEKL specific tetramer staining. **b.** CD8 and CD4 T cells were assayed for peptide antigen-specific release of IFNγ by intracellular cytokine staining and flow cytometric analysis. For all panels, * denotes a *p*-value < 0.05, two-sided *t*-test. Data are representative of 2 independent experiments. Error bars represent mean + SEM.

In the next studies we examined the influence of B cell direct presentation on the immune response to traditional intradermal DNA vaccination, through either B cell depletion or enrichment. Cross-presentation of antigen produced in bystander cells is thought to mediate a majority of the resulting cellular immunity resulting from DNA vaccination, with very little direct presentation naturally occurring [8, 18, 40]. Given the well documented ability of DCs to cross-present protein antigen to CD8 T cells [8, 18, 41, 42], we hypothesized that mice with intact DCs, but deficient in B cells, should still elicit an immune response following standard intradermal immunization, but might differ in the “quality” of that response given the lack of any direct presentation by B cells. Consequently, in the first study age-matched naïve wild type C57BL6 and B cell-deficient μMT mice were administered four weekly doses of 100μg of psOVA. One week after the final immunization, splenocytes from the animals were assayed for antigen-specific immune responses by tetramer binding and intracellular cytokine staining. As seen in Figure 7a, both wild type and μMT mice displayed equivalent frequencies of antigen-specific CD8 T cells as measured by SIINFEKL tetramer staining. However, wild type mice demonstrated significantly greater secretion of IFNγ to the SIINFEKL (CD8) and ISQAVHAAHAEINEAGR (‘323- 339′, CD4) epitopes by CD8 and CD4 T cells respectively. These data suggest that B cell mediated direct presentation of plasmid DNA after intradermal injection might affect the generation of Th1-biased cellular immunity.

**Figure 8:**
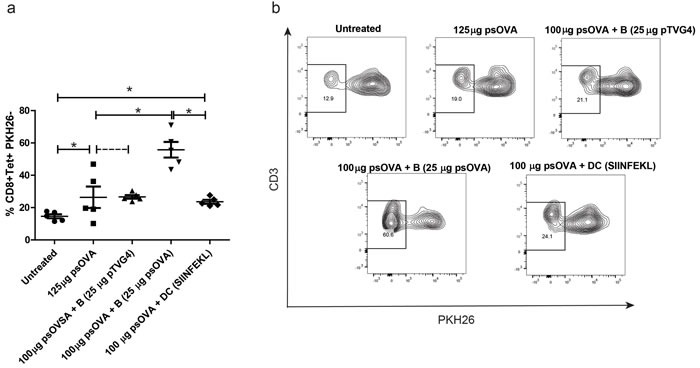
Increased direct presentation by plasmid treated B cells, and not cross presentation by DCs, further enhances DNA vaccine-induced CD8 T cell responses Age matched C57BL6 wild-type mice received PKH26-labeled OT-I splenocytes by adoptive transfer. One day later, mice were divided into groups (*n* = 5/group) and treated intradermally with either 125μg psOva, 100μg psOva + 10^6^ B cells pre-loaded with 25μg pTVG4, 100μg psOva + 10^6^ B cells pre-loaded with 25μg psOVA, or 100μg psOva + 10^6^ DCs pre-loaded with 2μg SIINFEKL peptide. One week later PKH dilution on SIINFEKL tetramer+ CD8 T cells from spleens was measured by flow cytometry. **a.** Frequencies of PKH26- CD8 T cells as measured by flow cytometry. Each data point represents one animal for each group. **b.** Shown is a representative flow dot-plot of PKH dilution for different treatment groups. For all panels, * denotes a *p*-value < 0.05, two-sided *t*-test. Data are representative of 2 independent experiments. Error bars represent mean + SEM.

In the next study we sought to evaluate the effect of increasing plasmid DNA direct presentation by B cells on DNA vaccine immunogenicity. Briefly, OT-I splenocytes were labeled with PKH26 and adoptively transferred into age-matched naïve C57BL6 mice. One day later, mice were divided into groups and administered DNA encoding ovalbumin alone, or with plasmid-treated B cells, or SIINFEKL peptide-treated DCs. One week later SIINFEKL tetramer^+^ CD3+CD8+ T cells were evaluated for proliferation by loss of PKH26 staining. As shown in Figure 8, augmenting traditional DNA vaccination with direct presenting B cells greatly increased proliferation of antigen specific CD8 T cells *in vivo*. Additional cross-presentation with peptide-loaded DCs did not have this effect.

## DISCUSSION

Vaccination with plasmid DNA is simple, safe, and economical. The ease with which plasmid DNA can be transported and mass-produced further highlights its potential as a vaccination approach for global health threats [8]. The discovery that simple intramuscular or intradermal inoculation of plasmid DNA elicited functional T cell immunity led to their exploration as anti-tumor vaccines [8, 12, 43]. In spite of remarkable preclinical efficacy against multiple solid tumor types, DNA vaccine trials in humans have been characterized by poor immunogenicity [8, 44, 45]. This difference between preclinical models and clinical studies has been suggested to be due to poor *in vivo* transfection efficiency and the resultant low antigen/body mass ratio [8]. Consequently, efforts to potentiate these vaccines have focused on increasing the antigen expression *in vivo*, both by increasing DNA delivery through electroporation or particle bombardment type approaches, and by enhancing the plasmid vector for expression in a mammalian host through the employment of Kozak sequences, codon optimization, tissue-specific promoters, and so on [19-21, 23, 46-49]. While most of these methods greatly increase antigen expression *in vivo*, they are likely to significantly potentiate only cross-presentation of antigen produced in bystander cells [18, 19, 22]. A relatively unexplored strategy to augment DNA vaccine efficacy is by specifically increasing direct presentation - where the antigen is produced in and is presented by a professional APC after plasmid uptake. Advances in the innate immunity field have highlighted the importance of direct presentation and cell-intrinsic activation of the APC in eliciting strong adaptive immunity [35]. In fact, the success of genetic vaccination with *Listeria* vectors is thought to be due in part to their ability to directly infect CD8α^+^DCs [50]. While different APC types have been found to contain plasmid DNA after either injection or particle bombardment *in vivo*, the ability of these cells to act as *bona fide* direct presenters after plasmid DNA uptake remains unresolved [51-54]. In the current studies, we sought to identify professional APC types that are able to internalize plasmid DNA, encode the antigen under a commonly used CMV promoter, and expand cognate T cells. We found that: 1) APC subsets isolated from human PBMC spontaneously take up plasmid DNA; 2) only B lymphocytes encode detectable amounts of antigen mRNA; 3) B lymphocytes co-cultured with plasmid DNA are able to expand cognate primary tumor antigen-specific CD8 T cells *in vitro*;** 4) murine B cells, and not DCs, are able to prime antigen-specific CD8 T cells *in vitro* and *in vivo*;** 5) direct antigen presentation of plasmid DNA by murine B cells is sufficient to cause an anti-tumor effect *in vivo*;** 6) B cell-deficient mice have impaired Th1 T-cell responses to intradermal DNA vaccination; and 7) supplementing traditional DNA vaccination with plasmid antigen presenting B cells, and not peptide antigen presenting DCs, leads to a robust increase in cognate CD8 T cell proliferation *in vivo.* Together, these results highlight that B cells, and not DCs, are direct presenting cells for plasmid DNA following simple co-culture, and that increasing such B cell mediated direct presentation may be advantageous for increasing Th1-biased T-cell immunity, notably for anti-tumor vaccines.

In order to identify potential direct presenting cells for plasmid DNA, we investigated plasmid uptake, antigen production, and antigen-specific T cell expansion as rational steps in the process. While uptake of naked λ phage DNA and oligonucleotides by different primary cell populations (leukocytes, skeletal myocytes, and keratinocytes) has been previously reported by several investigators, we were interested in comparing plasmid DNA uptake exhibited by different subsets in whole human PBMC [33, 55-57]. In our studies, primary APC exhibited rapid association with naked plasmid DNA in serum free conditions. This is consistent with past studies of DNA uptake in mammalian cells, as reviewed by Wheeler *et al* [33]. Several routes and mechanisms of internalization (membrane bound adaptor proteins, receptor mediated endocytosis, and pinocytosis) have been suggested for naked DNA uptake by different cells [33], but data from our investigations suggest that plasmid uptake, at least in the B lymphocyte and DC subsets, is by pinocytosis. Our data are consistent with a previous report of plasmid DNA uptake in keratinocytes predominantly through pinocytosis [58].

We further found that only B lymphocytes, and not DCs or monocytes/macrophages, were able to efficiently encode antigen mRNA upon simple co-culture with plasmid DNA as detected by RT-PCR. Interestingly, we were able to detect antigen mRNA only in enriched B cell populations, and not in whole PBMC (data not shown). This suggests that B lymphocytes might be outcompeted by other phagocytic myeloid cell types for plasmid DNA uptake, thereby reducing the number of plasmid-containing B cells. This might further explain the low immunogenicity of DNA vaccines in human trials, with a majority of the plasmid DNA ingested by tissue resident DCs and macrophages being degraded after internalization. Our results with DCs are surprising, given the highest attention they have received as targets for DNA vaccines *in vivo* over the last two decades [14, 17, 30, 51, 59]. Our findings with B lymphocytes corroborate those of Filaci *et al*, who first described “spontaneous lymphocyte transgenesis (SLT)”, of B cells upon co-culture of peripheral blood lymphocytes with plasmid DNA, and their ability to encode plasmid antigens in a series of elegant studies [32, 34, 60]. However, our starting material of DC-enriched PBMC allowed us to further investigate non-functional plasmid uptake by both DCs and monocytes/macrophages and their potential role in influencing availability of plasmid DNA to B cells. Interestingly, we were unable to detect transgene protein in any of our experiments, in spite of detectable mRNA levels. This is probably indicative of the expected low “efficiency” of transfection by passive co-culture, where a majority of the internalized DNA is destroyed in lysosomes [56, 61]. Even in the SLT model, detectable levels of a fluorescent protein marker were observed only after a multiplexed magnetic enrichment of transgenic cells [32, 34].

While detection of protein by even sensitive molecular biology methods like ELISA requires a concentration of at least 3pg/mL, or at least 72264540 molecules/mL of the SSX2 protein, a single peptide-MHC complex is sufficient to elicit T cell recognition and a cytolytic T cell response [62]. We therefore next compared the ability of monocytes/macrophages, DCs, and B cells to present plasmid encoded antigen to expand cognate CD8 T cells. As expected from our findings with mRNA, only B cells were able to expand pre-existing tumor antigen-specific CD8 T-cell responses in multiple donors (Figure 2). Our findings are therefore novel, and demonstrate that human B cells are best suited for direct presentation of plasmid DNA.

Our data in human and murine models demonstrate that only B cells co-cultured with plasmid DNA, and not DCs, are able to prime and expand naïve antigen-specific T cells. Delivery of B cells co-cultured with plasmid DNA, but not DC co-cultured with plasmid DNA, elicited anti-tumor responses in mice as well. These results are similar to those reported by Gerloni *et al*, in which adoptive transfer of B cells co-cultured with a plasmid encoding an engineered chimeric immunoglobulin heavy-chain gene containing MHC-I epitopes under an IgG promoter elicited functional T cell immunity and protection from lethal virus challenge *in vivo* [34]. Parallel studies using DCs were not possible in that model, given that the plasmids used encoded epitope antigens under IgG promoters [32, 34, 63]. Moreover, the principle behind those studies was the production of “antigenized antibodies”, Ig molecules containing T and B cell epitopes in their CDR2 and CDR3 variable regions. Secretion of such proteins by transgenic cells would lead to efficient Fc receptor mediated internalization and cross presentation in other APCs. In contrast, all our studies were conducted with plasmid DNA constructs encoding full length antigens under ubiquitous viral promoters, those that are more representative of traditional DNA vaccines as tested in human trials to date [25, 44, 45]. Our data thereby represent, to our knowledge, the first demonstration of B cell-mediated induction of functional anti-tumor immunity upon co-culture with plasmid DNA, as well as their first comparison with DCs in this setting.

Interestingly, we observed a loss of antigen-specific CD8 T cell cytokine secretion and CD4 T cell help in B cell deficient μMT mice, in spite of equivalent tetramer binding. These data suggest a potential role for direct presentation by B cells, and are consistent with a previous report by Castiglioni *et al,* in which they demonstrated that B cell presentation to CD8 T cells after transgenesis *in vitro* was dependent on CD4 T cell help [64]. Our data further correspond to findings that B cells in draining lymph nodes and bone marrow express plasmid encoded antigen and can influence CD8 T cell memory after DNA vaccination [53, 54]. Our results are in contrast with two previous reports describing equivalent T-cell responses upon DNA vaccination in B cell deficient mice [19, 65]. However, these discrepancies are possibly due to the employment of the gene-gun method of inoculation in those studies, where the predominance of cross-presentation as the primary mechanism of antigen presentation has been well documented [19, 22]. In separate experiments, we supplemented traditional DNA vaccination with either direct presentation by B cells or cross presentation by DCs. Interestingly, only increasing direct presentation through the use of B cells improved antigen specific CD8 T cell proliferation *in vivo*, suggesting that sufficient engagement of both direct presentation (by transfer of co-cultured B cells) and cross presentation (by traditional vaccination) leads to enhancement of DNA vaccine efficacy. This may be potentially due to greater induction of CD4 T cell help by direct presenting B cells, a limitation of cross presentation pathways.

Our results here indicate that B cells, and not DCs, are best suited for presentation of plasmid DNA encoded antigens upon passive uptake. These data are seemingly in discord with several seminal studies describing the isolation and *ex vivo* T cell stimulatory capacity of a small number of antigen- or antigen mRNA-containing DCs isolated from either skin explants or draining lymph nodes after DNA injection, electroporation, or gene-gun administration *in vivo* [14, 16, 17, 51, 66]*,* along with one report even describing direct presenting macrophages [15]. However, only one of these reports demonstrated the isolation of plasmid DNA in DCs from draining lymph nodes after vaccination [16]. The other reports used either rapidly degraded antigen, presence of mRNA, or non-secreted proteins as indicators for direct transfection of DCs *in vivo*. We now know that mRNA, intracellular protein, and even peptide loaded MHC complexes can be transferred to APCs by several mechanisms, most notably *via* extracellular vesicles [67, 68]. Moreover, these data might be a reflection of the inherent transfection capacity of gene-gun, electroporation, or even injection pressure during DNA administration *in vivo,* where bypassing of degradation pathways and nuclear transport could be more likely [33, 59]. It is also conceivable that there exist rare direct presenting Langerhans' or dermal DC sub-populations distinct from FLT3-L derived conventional DCs or monocyte derived DCs used in our studies. These cells are, however, less amenable to isolation, expansion, or large scale manipulation or targeting *in vivo,* when compared to B lymphocytes. Gene expression in, and *ex vivo* stimulatory capacity of, isolated DCs notwithstanding, several subsequent reports investigating the direct role of DCs in generation of T cell immunity through the use of DC/macrophage promoters, and/or bone marrow chimaeras have concluded that antigen expression in DCs *in vivo* is of little significance in *de novo* DNA vaccination immunogenicity, in spite of their T cell stimulatory capacity *ex vivo* [18-21]. These observations, combined with the data from our studies, indicate that uptake by DCs and other myeloid lineage cells might in fact be deleterious to DNA vaccine efficacy following passive transfer of DNA, with a majority of the plasmid being degraded intracellularly. As such, an interesting approach to study the effect of these non-presenting APC types on DNA vaccine immunogenicity might involve chemo-attraction away from the site of immunization to allow greater “useful” transfection of bystander cells and trafficking of plasmid molecules to B cell rich regional lymph nodes [53, 54, 69].

In summary, our data demonstrate that B cells, and not DCs, are optimal direct presenters of plasmid DNA when delivered by passive co-culture. They further highlight the potential of B cell-mediated direct presentation as a novel avenue for investigating improvements to DNA vaccination. Given their relative abundance, easy manipulation, and non-adherent culture characteristics, therapeutic B cell delivery, administration of B cell chemo-attractants and B cell specific adjuvants, as well as active targeting of plasmid DNA to B cells are exciting future directions for the field of DNA vaccinology.

## MATERIALS AND METHODS

### Donor blood samples

Peripheral blood mononuclear cells (PBMC) were previously collected from individuals with prostate cancer who provided written informed consent under IRB-approved protocols (NCT00582140 and NCT00849121). Samples were cryopreserved in 90% fetal calf serum and 10% DMSO, and stored in liquid nitrogen, until use.

### *In vitro* plasmid uptake and trafficking studies

Monocyte-derived DC were prepared as previously described [70]. Briefly, the adherent fraction from whole PBMC was isolated after 2h culture in serum-free AIM-V media (Thermofisher, Carlsbad, CA) and careful removal of non-adherent cells. Adherent mononuclear cells were then cultured in the presence of 10ng/mL IL-4 and 20ng/mL GM-CSF for 6 days to cause differentiation into CD11c^+^CD14^−^HLA-DR^+^CD45RO^+^ dendritic cells (> 80% by flow cytometry, data not shown). Fluorescently-labeled plasmid DNA was prepared either using the Cy5 Label IT^®^ intracellular nucleic acid localization kit (Mirus Bio, Madison, WI) as per the manufacturer's instructions, or by labeling with a bis-peptide nucleic acid (bis-PNA) probe (Panagene, Korea): Alexa 647-O-ttt tct cct-ooo-tjj tjt ttt-KK as previously described [71].

Cryopreserved human PBMC were used alone or combined with autologous immature monocyte-derived DC. Cells were washed twice in Ca/Mg free PBS, and cultured in the presence of 2μg/mL fluorescently-labeled plasmid DNA (pTVG4 construct, previously described [72]), 10μg/mL unlabeled plasmid DNA, 10μg/mL FITC Dextran (Sigma-Aldrich, St. Louis, MO), or 1:1000 FITC Latex (Cayman chemical company, Ann Arbor, MI) for 1h at different temperatures. Cells were then washed twice in PBS, labeled with antibodies specific for surface proteins CD3 (17A2, BD Biosciences, San Diego, CA), CD19 (HIB19, eBioscience, San Diego, CA), CD11c (N418, Tonbo, San Diego, CA), CD14 (M5E2, BD Biosciences), and Ghost live-dead 780 (Tonbo), and analyzed for plasmid associated fluorescence by either conventional flow cytometry (BD LSR Fortessa) or multi-spectral imaging cytometry at 40 or 60X magnifications (Amnis Imagestream X). For trafficking experiments, positively enriched cell subsets were incubated with plasmid DNA for 1h initially, followed by culture in RPMI/10% AB serum media (Valley Biomedical, Winchester, VA) in 12-well dishes (Corning, Vienna, VA). Samples were further fixed and permeabilized using Cytofix/Cytoperm (BD Biosciences), followed by staining with anti-CD107a/LAMP (H4A3, BD Biosciences) or Hoechst 33342 (Thermofisher) prior to analysis. All analysis was performed with the IDEAS (version 6.0) software (Amnis/EMD Millipore). Specifically, at least 100 in-focus images were used to conduct internalization (RawMaxPixel feature) and co-localization (BrightDetailSimilarity feature) analysis using the built-in analysis programs.

To assess degradation of plasmid DNA in the different cell types, enriched APC subsets were isolated using a PE positive selection kit (Stemcell, Vancouver, Canada). Each cell subset at 10^7^ cells/mL was incubated with 25μg/mL pTVG4 in PBS, with gentle shaking every 15 minutes. After 1h, cells were cultured at 2×10^6^ cells/mL in RPMI/10%AB media for up to 72 hours. Cells were then incubated with 10μg/mL DNaseI for 3h to digest any DNA that was not internalized. Total cell nucleic acid was then collected using the Zymo Prep ZR Duet kit^®^ (Zymo Research Corporation, Tustin, CA). Equal amounts of nucleic acid between the two time points were used to transform highly competent DH5α cells (Zymo Mix&Go Cells^®^, Zymo Research Corporation) using standard methods, plated on kanamycin-containing selection agar plates, and colonies were enumerated one day later.

### Human *in vitro* co-culture experiments

APC subsets were enriched using PE-labeled antibodies specific for either CD19, CD14, or CD11c as per the manufacturer's protocol (Stemcell). Cells were then washed in PBS, and cultured for 1h at 10^6^ cells/mL in the presence of 25μg/mL plasmid DNA (either empty vector pTVG4, or plasmids encoding SSX2 (pTVG-SSX2), Ovalbumin (psOVA), or EGFP (pEGFPc1)) with gentle shaking every 15 minutes. After 1h, cells were transferred to RPMI/10% AB media and cultured for 18h at 2-3*10^6^ cells/mL. For RNA detection experiments, total cell RNA was collected using the Zymo Direct-Zol^R^ RNA extraction kit (Zymo Research), and one step RT-PCR was performed using the SuperScript III One-Step RT-PCR system (ThermoFisher) using the following primers: AAGCAGGCAGAGAGGTGGTA (Ova 5′), GAATGGATGGTCAGCCCTAA (Ova 3′), AAGCTGACCCTGAAGTTCATCTGC (EGFP 5′), TCCAGCAGGACCATGTGATC (EGFP 3′), CATCTTCCTCAGGGTCGCTGATCTC (SSX2 5′), CCACAAAATGATGGGAAAGAGCTGTG (SSX2 3′). For quantitative PCR experiments, total cDNA was prepared using the iScript cDNA synthesis kit (Bio-Rad, Irvine, CA) and qPCR was performed using the Sso Fast Eva Green qPCR kit (Bio-Rad) with primers as above.

For tetramer expansion studies, equal numbers (2-4×10^6^) of negatively selected autologous T cells (Human T cell enrichment kit, Stemcell) were added to each APC culture prepared as above, in RPMI/10% AB, followed by addition of 0.5ng/mL IL-1β and 10U/mL of IL2 on day 2. On day 7, cells were stained with anti-CD3 (17A2, BD Biosciences), anti-CD4 (OKT4, eBioscience), anti-CD8 (OKT8, eBiosceince), and Live-Dead Ghost 780 (Tonbo), along with tetramers specific for the p41 or p103 peptide epitopes (NIH Tetramer Core Facility, Emory University), and analyzed by flow cytometry.

### Mice and cell lines

HLA-A2.01/HLA-DRI expressing, murine MHC class I/II knockout, mice (HHDII-DR1) on a C57BL6 background were obtained from Charles River Labs courtesy of Dr. Francois Lemonnier (Institut Pasteur, Paris, France). Wild-type C57BL6 mice and corresponding age- and sex-matched B cell deficient μMT (Ighm^tm1Cgn^) mice were obtained from Jackson Laboratories (Bar Harbor, Maine). OT-II splenocytes were a kind gift of Dr. Matyas Sandor. OT-I splenocytes were collected from heterozygous OT-I mice obtained from Jackson Laboratories. Mice were maintained under aseptic conditions and all experiments were conducted under an IACUC-approved protocol.

The A2/sarcoma cell line expressing SSX2 was generated as previously described [39].

### Murine co-culture studies

Primary B cells were obtained from mouse spleens by PE selection for CD19 as above. Primary dendritic cells were similarly obtained by positive selection for CD11c from spleens of mice previously inoculated with a B16 tumor cell line transduced to secrete FMS like Tyrosine kinase 3 ligand (Flt3-L). Use of the B16/Flt3-L cell line allowed the isolation of primary DCs generated *in vivo,* as previously described [50]. The B16-Flt3-L cell line was a kind gift from Dr. John-Demian Sauer.** After enrichment, each APC subset was cultured at 10^7^ cells/mL in PBS in the presence of 25μg/mL of plasmid, with brief shaking every 15 minutes for 1h. Cells were then transferred to RPMI/10% FCS (Thermofisher) for overnight culture.

For *in vitro* expansion studies, primary T cells were negatively selected from naïve OT-I and OT-II splenocytes using the mouse T cell isolation kit (Stemcell), labeled with PKH26 (Sigma Aldrich) according to the manufacturer's instructions, and added to APC cultures on day 2. Five days later, CD4 (GK1.5, BD Biosciences) and CD8 T (53-6.7, BD Biosciences) cells were analyzed for loss of PKH26 staining by conventional flow cytometry.

### *In vivo* immunizations and cell transfers

In all studies, mice in the DNA immunization group were administered 100μg of plasmid DNA intradermally in the ear pinna, as previously described [39, 73]. In studies involving B cell-deficient mice, female, 8-10 week old wild-type C57/BL6 mice and matched B cell-deficient μMT mice were given 4 weekly doses of plasmid encoding full length ovalbumin (psOVA or pCI-neo-sOVA, Addgene, MA). pCI-neo-sOVA was a gift from Maria Castro (Addgene plasmid #25098).

For cell transfer experiments, plasmid incubated APC cultures were prepared as described above, washed thoroughly with PBS after 18h of culture in RPMI/10% FCS, and injected intradermally in the ear pinna. 100μg of empty plasmid vector was injected in the opposite ear to control for DNA adjuvant effect. In groups receiving conventional DNA immunizations, 100μg of plasmid DNA alone was injected intradermally in the ear pinna, as previously described [39, 73]. Splenocytes from immunized mice were collected 1-2 weeks post treatment and analyzed for antigen-specific immune responses by intracellular cytokine staining as previously described [73]. Briefly, splenocytes were stimulated with the relevant peptide for 2h, followed by treatment with monensin (GolgiStop, BD Biosciences) for 4h. Cells were then surface stained as above, followed by fixation, permeabilization (Cytofix/Cytoperm, BD Biosciences) and cytokine staining: IFNγ (XMG1.2; BD Biosciences), TNFα (MP6-XT22; BD Biosciences), IL2 (JES6-5H4; eBioscience).

For tumor studies, age-matched mice were first inoculated with 5*10^4^ syngeneic SSX2 expressing sarcoma cells in 50% high concentration LDEV-free Matrigel (BD Biosciences) followed by biweekly immunization or APC transfer treatments beginning day 2 after tumor implantation. Tumor volume was measured in cubic centimeters according to the formula: (π/6)(long axis)(short axis) [39]. To assay for CD8 T cell infiltration, tumors obtained at necropsy were digested in media containing 1mg/mL collagenase and 20μg/mL DNase-I (Sigma, St. Louis, MO) for 3 hours at 37°C, and passed through a 100-μm screen to obtain a single cell suspension. This cell suspension was stained with anti-CD3 (17A2; BD Biosciences), anti-CD8 (53-6.7; BD Biosciences), and live-dead Ghost 780 (Tonbo). Immunohistochemistry for CD8α was performed on FFPE tumor tissue after antigen retrieval in citrate buffer (pH 6.0) prior to staining with a primary rat-anti-mouse CD8α antibody (4SM15; eBioscience) and a secondary anti-rat IgG (MP-444; Vector, Burlingame, CA). Standard development with DAB and hematoxylin counter-staining was performed.

## SUPPLEMENTARY MATERIALS FIGURES AND TABLE


