# Supplementary material for: B lymphocytes as direct antigen-presenting cells for anti-tumor DNA vaccines
Source: Oncotarget. 2016 Sep 21;7(42):67901–18. doi: 10.18632/oncotarget.12178 (PMC5356528; doi:10.18632/oncotarget.12178)
Supplement: Supplementary file 1 [file oncotarget-07-67901-s001.pdf]

B lymphocytes as direct antigen-presenting cells for anti-tumor DNA vaccines

Supplementary Material

Supplemental Table 1: Primary human B cells encode plasmid mRNA and mediate expansion of cognate antigen specific CD8 T cells upon plasmid DNA treatment.

Donor 1	**Attempt 1**	**Attempt 2**	**Attempt 3**	
**CD11c/ pTVG4**	0.769	0.45	0.31	
**CD11c/ SSX2**	0.691	0.514	0.41	
**CD14/pTVG4**	0.18	0.43	0.29	Baseline 0.74
**CD14/SSX2**	0.306	0.36	0.36	
**CD19/pTVG4**	0.7	0.61	0.68	
**CD19/SSX2**	2.1	1.38	1.54	

Donor 2	**Attempt 1**	**Attempt 2**	**Attempt 3**	
**CD11c/ pTVG4**	0.564	1.12	0.84	
**CD11c/ SSX2**	1.84	1.91	1.12	
**CD14/pTVG4**	1.29	0.84	1.64	
**CD14/SSX2**	2.34	2.04	1.92	Baseline 2.05
**CD19/pTVG4**	2.06	2.35	1.81	
**CD19/SSX2**	5.64	4.84	3.64	

Donor 3	**Attempt 1**	**Attempt 2**	
**CD11c/ pTVG4**	0.868	0.65	
**CD11c/ SSX2**	0.764	0.7	
**CD14/pTVG4**	0.238	0.51	Baseline 0.98
**CD14/SSX2**	0.84	0.79	
**CD19/pTVG4**	1.05	0.61	
**CD19/SSX2**	2.21	1.88	

Enriched APC subsets from HLA-A2+ donors with known tetramer responses to SSX2 (shown as baseline %) were treated with 25µg/mL empty vector control (pTVG4) or a plasmid encoding SSX2 (pTVG-SSX2) in the presence of autologous T cells. One week later, samples were assayed for p103-specific CD8 T cells by tetramer staining. Shown are the % of p103-specific tetramer+ events among all CD8+ T cells for each culture condition for replicate studies using 3 different donors.

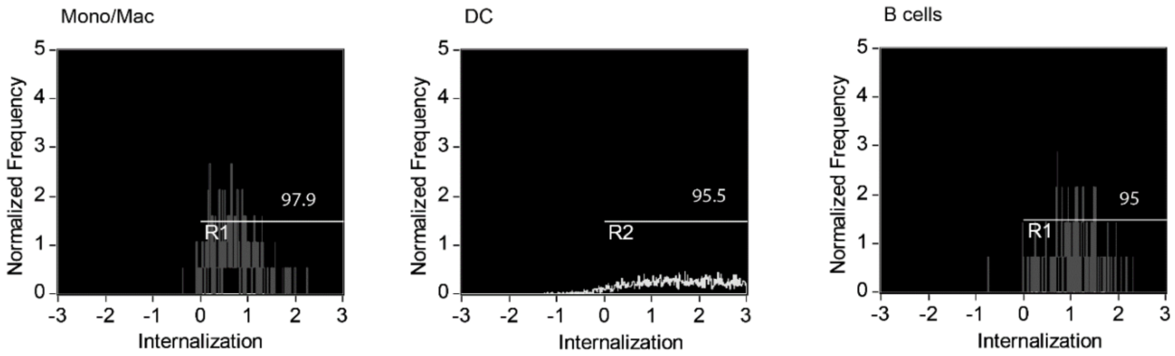

Figure S1. Plasmid Cy5 fluorescence internalization using imaging cytometry. Representative plots showing the internalization score distributions of plasmid associated Cy5 fluorescence on different APC populations from studies in Figure 3. A score ≥0 signifies the presence of Cy5 probe internal to the relevant cell surface marker (CD14, CD11c, or CD19 for monocytes, DCs, or B cells respectively). Plots were extracted from the IDEAS image analysis software.

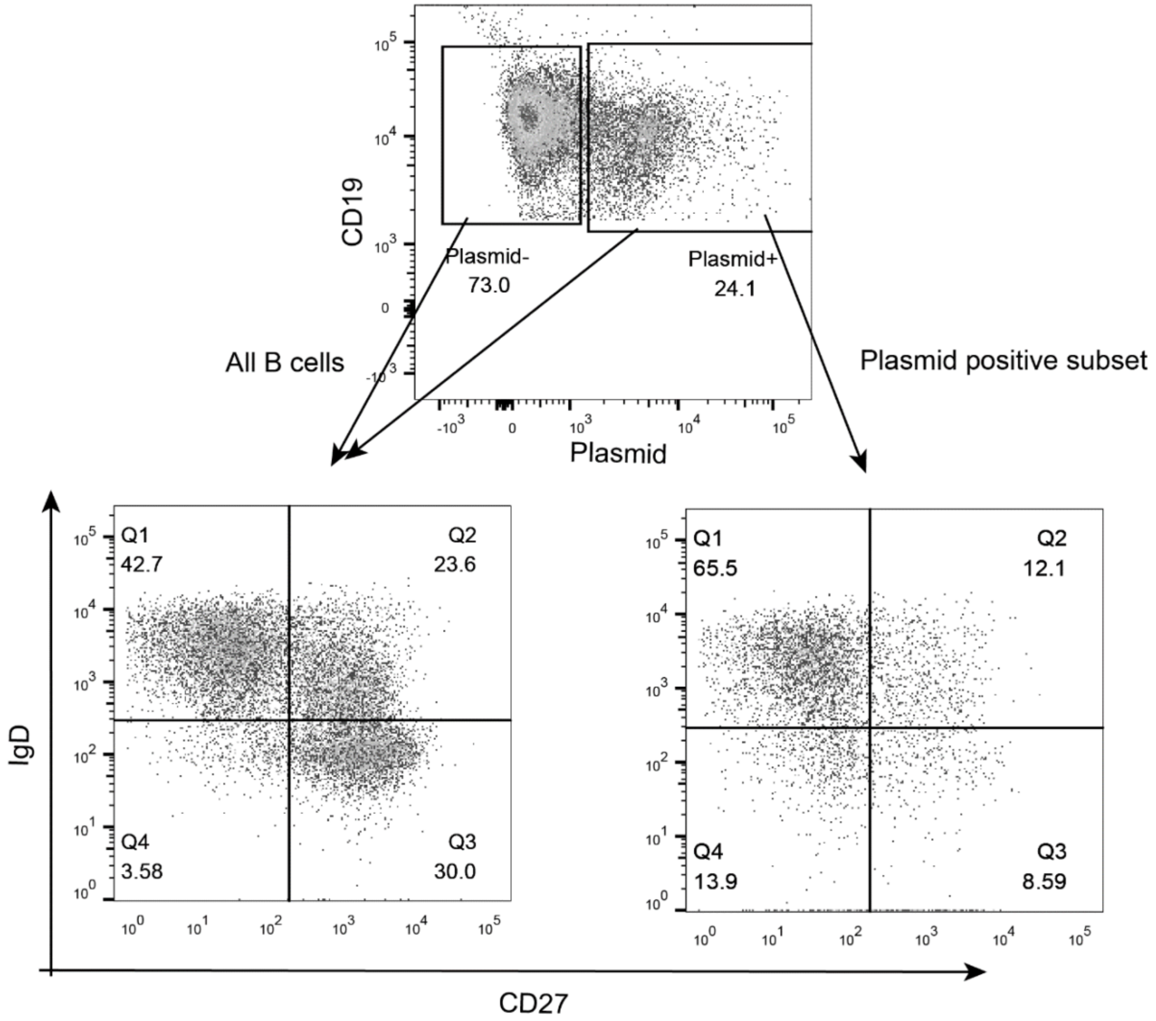

Figure S2. B cell mediated plasmid uptake occurs predominantly in the IgD$^+$CD27$^-$ subset. Cells were treated as in Figure 1B, along with additional staining for cell surface markers relevant to different B cell subsets prior to flow cytometry. Shown are flow data and the gating strategy associated with the analyses on a sample from one representative donor.

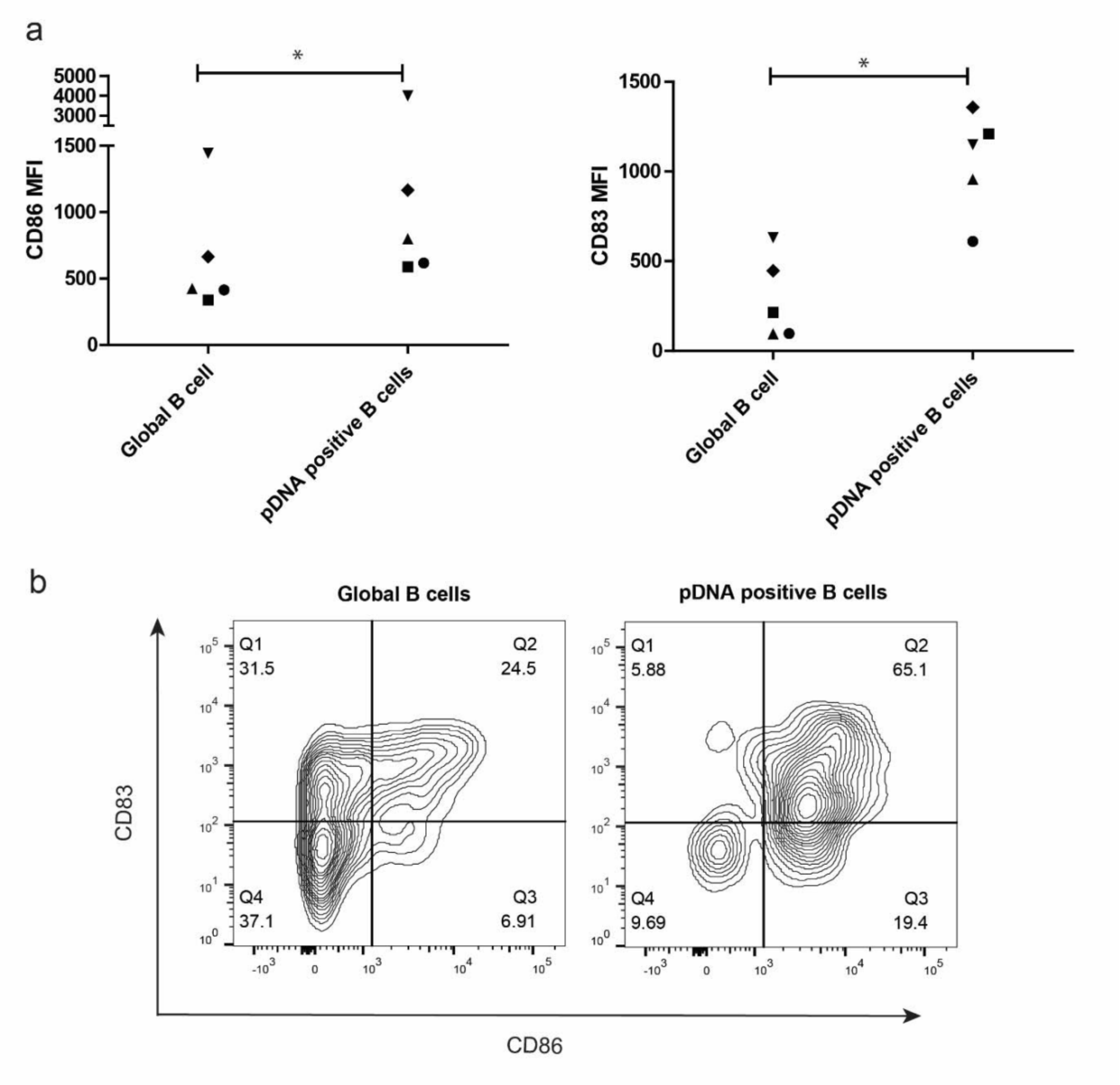

Figure S3. Plasmid uptake results in activation of B cells. Cells were treated with fluorescently labeled plasmid DNA as in Figure 1B, and incubated for 24h. Additional staining for markers of activation and costimulation was performed prior to flow cytometric analysis. (a) MFI of CD86 (left) and CD83 (right) staining on the non-plasmid containing B cell population (global B cell) and the plasmid positive subset. Each symbol represents one donor. (b) Representative flow cytometry contour plots showing CD83 and CD86 levels on either all non-plasmid-containing B cells (left) or the plasmid-positive subset (right). For panel A, * denotes a p-value <0.05, two-sided non parametric paired t-test.
